# Potential involvement of beta-lactamase homologous proteins in resistance to beta-lactam antibiotics in gram-negative bacteria of the ESKAPEE group

**DOI:** 10.1186/s12864-024-10410-2

**Published:** 2024-05-22

**Authors:** Joyce de Souza, Alexandre Zanatta Vieira, Hellen Geremias dos Santos, Helisson Faoro

**Affiliations:** 1 Laboratory for Applied Science and Technology in Health, Carlos Chagas Institute, FIOCRUZ, Paraná, 81350-010 Brazil; 2 Carlos Chagas Institute, FIOCRUZ, Paraná, 81350-010 Brazil; 3 Department of Microbiology, Infectious Disease and Immunology, CHU de Quebec Research Center, University Laval, Quebec, QC G1V 0A6 Canada

**Keywords:** Beta-lactamase, homologous protein, ESKAPEE, resistance, beta-lactam antibiotics

## Abstract

Enzymatic degradation mediated by beta-lactamases constitutes one of the primary mechanisms of resistance to beta-lactam antibiotics in gram-negative bacteria. This enzyme family comprises four molecular classes, categorized into serine beta-lactamases (Classes A, C, and D) and zinc-dependent metallo-beta-lactamases (Class B). Gram-negative bacteria producing beta-lactamase are of significant concern, particularly due to their prevalence in nosocomial infections. A comprehensive understanding of the evolution and dissemination of this enzyme family is essential for effective control of these pathogens.

In this study, we conducted the prospecting, phylogenetic analysis, and in silico analysis of beta-lactamases and homologous proteins identified in 1827 bacterial genomes with phenotypic data on beta-lactam resistance. These genomes were distributed among *Klebsiella pneumoniae* (45%), *Acinetobacter baumannii* (31%), *Pseudomonas aeruginosa* (14%), *Escherichia coli* (6%), and *Enterobacter* spp. (4%). Using an HMM profile and searching for conserved domains, we mined 2514, 8733, 5424, and 2957 proteins for molecular classes A, B, C, and D, respectively. This set of proteins encompasses canonical subfamilies of beta-lactamases as well as hypothetical proteins and other functional groups. Canonical beta-lactamases were found to be phylogenetically distant from hypothetical proteins, which, in turn, are closer to other representatives of the penicillin-binding-protein (PBP-like) and metallo-beta-lactamase (MBL) families. The catalytic amino acid residues characteristic of beta-lactamases were identified from the sequence alignment and revealed that motifs are less conserved in homologous groups than in beta-lactamases. After comparing the frequency of protein groups in genomes of resistant strains with those of sensitive ones applying Fisher’s exact test and relative risk, it was observed that some groups of homologous proteins to classes B and C are more common in the genomes of resistant strains, particularly to carbapenems.

We identified the beta-lactamase-like domain widely distributed in gram-negative species of the ESKAPEE group, which highlights its importance in the context of beta-lactam resistance. Some hypothetical homologous proteins have been shown to potentially possess promiscuous activity against beta-lactam antibiotics, however, they do not appear to expressly determine the resistance phenotype. The selective pressure due to the widespread use of antibiotics may favor the optimization of these functions for specialized resistance enzymes.

**Supplementary Information:**

The online version contains supplementary material available at 10.1186/s12864-024-10410-2.

## Background

Antimicrobial resistance (AMR) is considered a major public health challenge globally. Worldwide, it is estimated that the number of deaths resulting from infections caused by resistant microorganisms was around 1.27 million in 2019, and could reach approximately 10 million in 2050 [1, 2]. Approximately 141,000 and 243,000 of these deaths were attributed to a combination of pathogen and resistance to third-generation cephalosporins and carbapenems, respectively [1]. The group of bacteria called ESKAPEE (*Enterococcus faecium*, *Staphylococcus aureus*, *Klebsiella pneumoniae*, *Acinetobacter baumannii*, *Pseudomonas aeruginosa*, *Enterobacter* spp. and *Escherichia coli*) plays a fundamental role in this scenario due to its high prevalence in nosocomial infections and increasing resistance to multiple drugs, especially among gram-negative bacteria of this group [3–5]. Gram-negative bacteria from the ESKAPEE group that are resistant to carbapenems or third-generation cephalosporins, especially in the case of Enterobacteriaceae, are considered a critical priority for the development of new antibiotics [5].

Beta-lactam antibiotics are a class of antimicrobials that have been widely used for over 70 years, mainly due to their safety, efficacy, and broad spectrum of activity. They are divided into different chemical classes—penicillins, cephalosporins, carbapenems, monobactams, and penems—which share as their main characteristic the presence of a beta-lactam ring in their structure. Gram-negative bacteria use enzymatic degradation facilitated by beta-lactamases as their main defense mechanism against beta-lactams. This presents a significant challenge in clinical settings as it limits treatment options for bacterial infections [6]. Local outbreaks of beta-lactamase-producing bacteria tend to spread to other regions, contributing to the global spread of organisms with very limited treatment options [7].

Beta-lactamases belong to the α/β-hydrolase family [8]. They were first identified in 1940, when was reported the observation of an enzyme capable of degrading penicillin [9]. Beta-lactamases are biochemically categorized according to the general characteristics of the catalytic site, being divided into two categories. Serine beta-lactamases (SBL) employ a serine in the active site to perform hydrolysis, while metallo-beta-lactamases (MBL) depend on one or two divalent zinc atoms (Zn^2+^) for this process [10]. Molecular classification, based on amino acid sequence, classified MBLs as class B, while SBLs are subdivided into classes A, C, and D [10, 11]. Furthermore, a functional classification distinguishes beta-lactamases according to substrate and inhibitor profiles [12, 13].

The number of variants described for beta-lactamases now exceeds 2770 and continues to increase rapidly [10]. At the same time, the amount of information available in public databases has shown exponential growth in recent decades, because of the advent of high-throughput DNA sequencing technologies. However, a significant fraction of genes from sequenced genomes are annotated as 'Hypothetical Proteins' or 'Conserved Hypothetical Proteins', due to the difficulty of functional characterization [14]. Hypothetical proteins lack functional annotations due to their lack of significant similarity to proteins or protein domains with characterized functions. However, these proteins fulfill biological functions whose identification relies on extensive molecular biology experiments and phenotypic analyses [15]. Consequently, numerous studies resort to utilizing databases and conducting phylogenetic analyses to infer the biological function of hypothetical proteins [16, 17]. In the context of antibiotic resistance, the selective pressure exerted by antimicrobial agents can induce rapid changes in protein sequence and function. This phenomenon complicates the characterization of potentially emerging resistance factors, which may exhibit latent promiscuous activity. Such sequences represent a reservoir of poorly characterized potential resistance genes, including beta-lactamases, where previously uncharacterized or hypothetical proteins may correspond to new variants.

In this context, enzymatic promiscuity emerges as an area of interest, because it is believed that latent activities of enzymes can lead to the emergence of new functions through the optimization of pre-existing activities. This is seen as a model of enzymatic evolution [18, 19]. The ability of proteins from the penicillin-binding-protein (PBP) and metallo-beta-lactamase (MBL) superfamilies to exhibit enzymatic promiscuity for the hydrolysis of beta-lactams has already been reported [18, 20]. However, little is still known about the possible presence and distribution of proteins with these functional characteristics in species of clinical relevance, as well as whether they can contribute to the manifestation of the resistance phenotype.

Given the significant mass of publicly available genomic data, as well as phenotypic information on antibiotic resistance, bioinformatics analyzes have great potential to assist in understanding the evolution and dispersion of beta-lactamases and this relation of beta-lactam enzymatic promiscuity. In the present work we carried out prospecting, phylogenetic analysis and in silico analysis of the functional potential of beta-lactamases and homologous proteins in public genomes of gram-negative bacteria from the ESKAPEE group. The use of a method of prospecting for homologous sequences and subsequent cluster analysis in relation to known variants helped to allocate proteins previously annotated as hypothetical and to identify groups that potentially contribute to the resistance phenotype presented by the organisms.

## Methodology

### Genome selection

The genome set was selected from the Bacterial and Viral Bioinformatics Resource Center (BV-BRC) database v.3.6.12 [21]. To obtain genomes of the gram-negative species within the ESKAPEE group that are resistant and susceptible to beta-lactam antibiotics, we filtered the total available bacterial genomes in the database (604,817) based on the Antimicrobial Resistant Phenotype ("AMR Phenotype") metadata. Selected genomes were limited to strains with resistance phenotypes determined experimentally in the laboratory (‘Laboratory Typing Method’ = MIC) for which the antibiotic concentration value (‘Measurement Value’), determined according to the Clinical and Laboratory Standards Institute standards (‘Testing Standard’ = CLSI), was available. Each selected genome is associated with at least one antibiotic. Genomes with assemblies above 100 contigs were eliminated, this filter was used as a quality standard, to eliminate genomes with very fragmented assemblies. The metadata and proteome associated with each genome were downloaded in record table (csv) and “fasta” formats, respectively.

### Prospecting for beta-lactamases and homologous proteins

Protein sequences of beta-lactamases from the four available molecular classes (A, B, C, and D) were retrieved from the Integrative Database of beta-Lactamase Enzymes [22]. These sequences were subjected to domain profiling using the NCBI Batch CD-Search based on the identifiers of conserved domains from the Pfam database [23]. All incomplete sequences were eliminated from the analysis in the conserved domain identification step. Sequences for which the Batch CD-Search result indicated non-specific hits or that the Pfam C- or N-terminal domain ends were incomplete were removed. A set of 601, 118, 148, and 52 sequences showed specific hits, respectively, for the class A identifiers (pfam13354—Beta-lactamase2), class B (pfam00753—Lactamase_B), class C (pfam00144—Beta-lactamase), and class D (pfam00905—Transpeptidase). Each set of sequences was individually aligned using the MAFFT v7.490 program [24]. Based on the alignment results, molecular profiles were created for each class of beta-lactamase to enable the prospecting of homologous proteins in the proteomes of the selected strains. These profiles were constructed using Hidden Markov Models (HMM) with the HMMsearch algorithm implemented in the HMMER v. 3.3.2 program [25]. Using NCBI Batch CD-Search, conserved protein domains were searched for in the prospected proteins. The identified proteins were filtered by the presence of the Pfam domain assigned to each class.

### Phylogenetic analysis

The homologous proteins prospected for each of the four classes of beta-lactamases were combined with the sequences used to build the HMM model. The redundancy of sequences in the dataset was removed using the CD-HIT v.8.1 program [26] applying a sequence identity threshold of 90%. Additionally, we conducted a literature search for studies that had performed the functional characterization of homologous proteins that could be acting as beta-lactamases. All sequences were checked for the presence of the complete beta-lactamase Pfam domain. Proteins with incomplete domains were discarded. Finally, for the sequence alignment and construction of the phylogenetic tree, only the regions corresponding to the Pfam domains of each protein were used. The resulting dataset was used in the construction of the phylogenetic trees. The alignment of the resulting sequence sets was performed using the MAFFT v7.490 program [24]. The alignment results were trimmed to remove positions with more than 95% gaps using the TrimAL v. 1.2.59 [27]. For phylogenetic analyses, the maximum likelihood method was used with 3000 ultrafast bootstrap replicates, implemented in the IQ-Tree v.1.6.12 program [28]. The display, annotation and export of tree images were carried out using the iTOL tool [29].

### Assessment of catalytic motifs

We identified, based on the literature, the catalytic motifs of each of the four classes: class A—S^70^xxK S^130^DN (KR^234^)(TS)G [30], class B—(HNQ)^116^xHxD H^**196**^ C^221^ H^263^ [31], class C—S^64^XXK Y^150^XN K^315^(S/T)G [32] and class D—S^70^xxK (YF)GN K^216^(TS)G [33]. The prospected proteins were grouped according to phylogenetic proximity and the positions corresponding to the catalytic motifs were selected from the alignment produced with the MAFFT v7.490 program [24]. The conservation of amino acid residues in the catalytic motifs was proven using the WebLogo v.3.7.12 program [34].

### Comparison of protein groups according to genomes resistance

The MIC for the beta-lactam antibiotics we analyzed varied from 0.015 to 256 mg/L. Due to the rare frequency of some MIC values (Supplementary Figure S1A), we categorized these values into three resistance phenotype groups: S) susceptible; R) resistant (MIC greater than or equal to 8 and less than 32 mg/L) and R2) resistant (MIC greater than or equal to 32 mg/L) (Supplementary Figure S1B). Fisher's exact test was applied as it is suitable for evaluating the association between two categorical variables represented in a contingency table, in this case, the three types of MIC (S, R, and R2) and the presence or absence of the protein group. Additionally, we performed a Post Hoc Pairwise Fisher Test, to compare the presence or absence of these groups between MIC categories. Since we tested three types of MIC, we corrected the *p*-value of Fisher's exact tests for multiple comparisons by applying False Discovery Rate correction (FDR). The test indicated an association for *p*-value according to FDR less than 5%. To quantify the relative frequency of protein presence in each MIC type and compare these frequencies between MIC categories, we estimated the relative risk, by considering the susceptible (S) MIC type as the reference category. Statistical analyses were all performed in R, using the stats v.4.3.2, rstatix v.0.7.1, and DescTools v.0.99.50 libraries.

## Results

### Genome selection and prospecting for beta-lactamases and homologous proteins

Data mining in the BV-BRC database returned a set of 1827 genomes with resistance phenotypes for different beta-lactam antibiotics (Supplementary Table S1). Regarding the distribution of genomes by species (Table 1), *K. pneumoniae* has the highest representation (45.1%), followed by *A. baumannii* (31.36%), *P. aeruginosa* (12.7%), *E. coli* (6.46%), and species of the *Enterobacter* genus (4.38%). A total of 9,165,389 proteins were retrieved from the selected genomes, of which 19,628 (0.21%) were identified as beta-lactamases or proteins homologous to beta-lactamases. The most abundant enzyme class is B, representing 44.49% of the sequences, followed by class C (27.63%), class D (15.07%), and class A (12.81%). Concerning the intraspecies distribution (Table 1), class B proteins were identified in a higher proportion in *K. pneumoniae*, *A. baumannii*, and *P. aeruginosa*, while for *E. coli* and *Enterobacter* spp., class C enzymes are in a higher proportion. When normalized by the number of genomes, *P. aeruginosa* and *E. coli* are the species with the highest and lowest number of prospected beta-lactamases and homologous proteins, respectively.

**Table 1:** Prospected genomes and proteins

Specie	# Genomes	# Proteins	Class A	Class B	Class C	Class D	Beta-lactamases/Genome
*K. pneumoniae*	824	9636	2050 (21.27%)	3933 (40.82%)	1753 (18.19%)	1900 (19.72%)	11.69
*A. baumannii*	573	5405	223 (4.13%)	2916 (53.95%)	1689 (31.25%)	577 (10.68%)	9.43
*P. aeruginosa*	232	3090	14 (0.45%)	1427 (46.18%)	1397 (45.21%)	252 (8.16%)	13.31
*E. coli*	118	852	145 (17.02%)	233 (27.35%)	330 (38.73%)	144 (16.90%)	7.22
*Enterobacter* spp.	80	645	82 (12.71%)	224 (34.73%)	255 (39.53%)	84 (13.02%)	8.06
	1827	19,628	2514	8733	5424	2957	

### Functional distribution

The functional classification of the prospected proteins, according to the annotation provided by the BV-BRC database, showed that, in addition to proteins classified as beta-lactamases, the sequence sets also included proteins from other functional groups and hypothetical proteins (Table 2).

**Table 2:** Functional distribution of prospected proteins using the HMM profile

Class	Protein description / Product	# Seqs
A	Class A beta-lactamase (EC 3.5.2.6)—SHV, CTX-M, TEM, KPC, LAP, CARB/PSE, PER, CARB/RTG, GES, OKP-B, LEN, VEB, IMI, OKP-A	2513
	Hypothetical protein	1
B	Metallo-beta-lactamase (MBL) superfamily protein	6150
	Hypothetical metal-binding enzyme	1593
	N-acyl homoserine lactone hydrolase	696
	Pseudomonas quinolone signal response protein (PqsE)	230
	Subclass B1 beta-lactamase (EC 3.5.2.6)—VIM, IMP, NDM, SPM	26
	Hypothetical protein	22
	Subclass B3 beta-lactamase (EC 3.5.2.6)	15
	Putative alkyl/aryl-sulfatase	1
C	Putative esterase	1841
	Class C beta-lactamase (EC 3.5.2.6)—ADC, PDC, BlaEC, ACT/MIR, CMY/CMY-2/CFE/LAT, DHA/MOR, FOX/TRU, CMH	1091
	D-alanyl-D-alanine-carboxypeptidase/endopeptidase AmpH	1020
	Beta-lactamase class C-like and penicillin binding proteins (PBPs) superfamily	1020
	Hypothetical protein	585
	UPF0214 protein YfeW (Putative D-alanyl-D-alanine carboxypeptidase)	55
	Polyketide synthase modules and related proteins	16
D	Peptidoglycan D,D-transpeptidase *Mrd*A (EC 3.4.16.4)	2643
	Class D beta-lactamase (EC 3.5.2.6)—OXA	314

The proteins prospected in the genomes were quantified according to the protein product annotations retrieved from the BV-BRC database

**Fig. 1:**
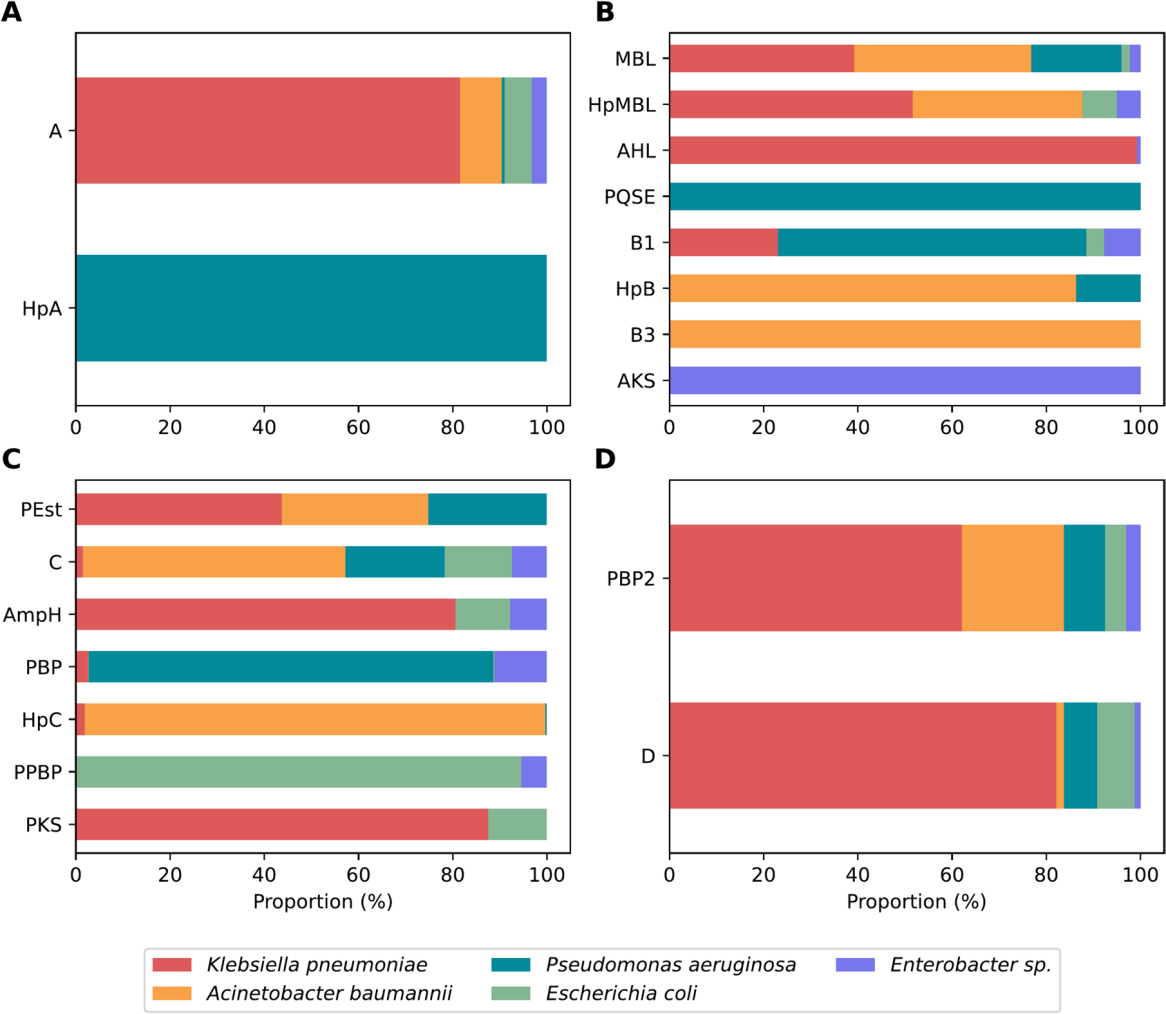
Distribution of proteins by species. The sequence sets corresponding to each class were grouped according to the description of the protein product as follows: **A** A—Class A beta-lactamases; HpA—Hypothetical proteins. **B** MBL—Metallo-beta-lactamases; HpMBL—Hypothetical metal-binding enzymes; AHL—N-acyl homoserine lactone hydrolases; PQSE—Pseudomonas quinolone signal response proteins; B1—Subclass B1 beta-lactamases; HpB—Hypothetical proteins; B3—Subclass B3 beta-lactamases; AKS—Putative alkyl/aryl-sulfatase. **C** PEst—Putative esterases; C—Class C beta-lactamases; AmpH—D-alanyl-D-alanine-carboxypeptidase/endopeptidase; PBP—Penicillin-binding proteins superfamily; HpC—Hypothetical proteins; PPBP—Putative penicillin-binding proteins superfamily; PKS—Polyketide synthase modules and related proteins. **D** PBP2—Peptidoglycan D,D-transpeptidase; D—Class D beta-lactamases

The class A set is quite homogeneous concerning the product, as the proteins are already annotated within the beta-lactamase families of this class, except for one hypothetical protein in *P. aeruginosa* (Fig. 1A). In the class B group, 88.92% of the proteins are hypothetical or only identified as belonging to the metallo-beta-lactamase (MBL) superfamily. The B3 subclass beta-lactamases appear in *A. baumannii*, and the B1 subclass in the other species (Fig. 1B). Additionally, three other protein groups were identified in the sequence set: N-acyl homoserine lactone hydrolases (AHL) in *K. pneumoniae* and *Enterobacter* spp., Pseudomonas quinolone signal response proteins (PqsE) in *P. aeruginosa*, and a Putative alkyl/aryl-sulfatase (AKS) in *Enterobacter* spp.

For the class C set, 32.71% of the sequences were classified as Putative esterases, followed by sequences related to the classic families of beta-lactamases in this class, identified in all the species included in this study (Fig. 1C). Additionally, two other functional groups, identified as D-alanyl-D-alanine carboxypeptidases and Polyketide synthases (PKS), along with hypothetical proteins or those only identified as belonging to the Penicillin-binding protein (PBP) superfamily, complete the functional distribution of this group.

Class D beta-lactamases belong to the OXA family and are distributed across all the species studied (Fig. 1D). Another sequence group identified as part of this class corresponds to Peptidoglycan D,D-transpeptidases, encoded by the *mrdA* gene, which produces penicillin-binding protein 2 (PBP2) and is also distributed among all the species included in this study.

### Phylogenetic relationship between classical *beta*-lactamases and homologous proteins

The prospecting for proteins homologous to beta-lactamases identified many proteins with different functions. These homologous enzymes represent a significant proportion of beta-lactamases from classes B and C, while they do not appear to have a significant impact on classes A and D. Thus, to investigate the relationships between classical beta-lactamases and homologous proteins, we constructed phylogenetic trees for each class, including these two sequence sets.

**Fig. 2:**
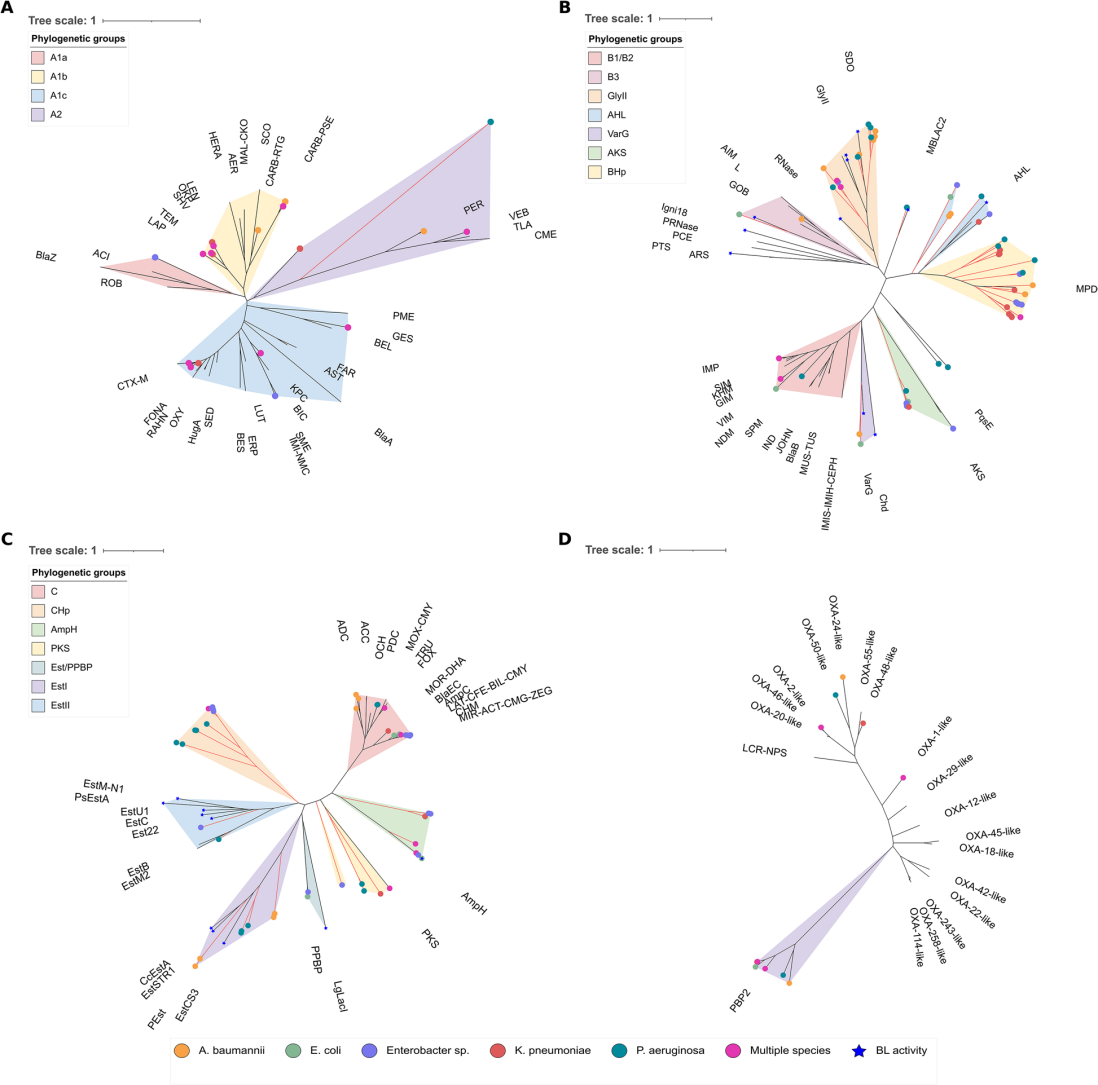
Phylogenetic analysis of beta-lactamases and homologous proteins. Phylogenetic tree constructed using the Maximum Likelihood (ML) method. The scale represents one amino acid change per site per million years. The orange branches represent enzymes without functional classification, composed of sequences whose description includes the words hypothetical, putative or superfamily. Branches without species identification represent sequences from the Integrative beta-lactamase database or other enzyme families included in the phylogeny. **A** Phylogenetic tree for class A beta-lactamases (pfam domain 13,354) and homologous proteins that includes 19 proteins representing clusters prospected from the genomes and another 31 representatives of beta-lactamases families. **B** Phylogenetic tree for beta-lactamase class B (domain pfam00753) which includes 60 proteins representing clusters prospected from the genomes and 42 proteins representing families of beta-lactamases and different functional groups of Metallo-hydrolases—ARS: arylsulfatase, PTS: phytase, PCE: Phosphorylcholine esterase, PRNase: putative ribonuclease, Igni18: promiscuous ancestral enzyme, Hp: hypothetical protein, RNase: ribonuclease, GlyII: glyoxalases II, SDO: sulfur dioxygenase, MBLAC2: human metallo-beta-lactamase, AHL: N- acyl homoserine lactone hydrolases, MDP: methy-parathion hydrolase, PqsE: Pseudomonas quinolone signal response protein, AKS: alkylsulfatase, Chd: Chlorothalonyl dehalogenase, VarG: putative metallo-beta-lactamase. **C** Phylogenetic tree for beta-lactamase class C (domain pfam00144) which includes 49 proteins representing clusters prospected from the genomes and 19 proteins representing families of beta-lactamases and different functional groups of Serine-hydrolases—AmpH: carboxylpeptidase, PKS: polyketide synthase, PPBP: Putative penicillin binding proteins superfamily. **D** Phylogenetic tree for class D beta-lactamase (domain pfam00905) which includes 11 proteins representing clusters prospected from the genomes and other 13 families of beta-lactamases—PBP2: Peptidoglycan D, D-transpeptidase

The phylogenetic tree of class A (Fig. 2A), as suggested previously by functional analysis, displays canonical families of class A beta-lactamases and one hypothetical protein. The prospected proteins from the genomes are phylogenetically divided into seven main groups: 1) CARB/PSE/RTG/SCO, 2) LEN/OKP/SHV/TEM/LAP, 3) ACI/BlaZ/ROB, 4) CTX-M, 5) KPC/BIC/SME/IMI/NMP, 6) GES/BEL, and 7) PER/VEB/TLA/CME. The PER/VEB/TLA/CME clade represents the families of subclass A2, which are distinct from the other A1 families [30]. The hypothetical protein in the class A set is phylogenetically distant from the other families; however, it originates from the A2 clade. A similar situation was observed in the class D tree (Fig. 2D), which represents the canonical families of class D beta-lactamases plus the functional group of PBP2s. The PBP2 proteins formed two species-specific divided groups, where the proteins from *K. pneumoniae*, *E. coli*, and *Enterobacter* spp. are in one clade, and those from *P. aeruginosa* and *A. baumannii* are in another. Class D beta-lactamases are distant from PBP2s and show phylogenetic peculiarities among themselves. The prospectively identified OXA family proteins from the genomes belong to four groups: 1) OXA-1-like, 2) OXA-55/48-like, 3) OXA-24/50-like, and 4) OXA-2/46/20-like.

Unlike the beta-lactamases of classes A and D, the sequence sets of beta-lactamases from classes B and C have a large number of proteins classified as hypothetical or with other functions. To assess the potential role of these proteins as beta-lactamases, we searched the literature for non-beta-lactamase proteins that exhibited experimentally determined beta-lactamase activity. The sequences of these proteins with beta-lactamase activity (Supplementary Tables S2 and S3) were included in the phylogenetic trees of the beta-lactamases from classes B and C. Class B beta-lactamases have two distinct phylogenetic origins, one that encompasses the B1/B2 subclasses and another the B3 subclass. In the analyzed genomes we identified enzymes from the B1 subclass (IMP, VIM, NDM and SPM families) and B3. Most of the hypothetical proteins and those classified only as MBL superfamily do not belong to the phylogenetic groups of the classical beta-lactamases families. Interestingly, many of them are close to families of proteins with other functions, but which also have beta-lactamase activity to some degree. These protein families are part of four main groups: 1) Ribonucleases (RNase), Glyoxalases II (GlyII) and Sulfur Dioxygenases (SDO), 2) Human metallo-beta-lactamases (MBLAC2), 3) Lactone hydrolases (AHL) and 4) Putative MBL (VarG) and Dehalogenases (Chd). The groups of proteins Pseudomonas quinolone signal (PqsE), Alkyl sulfatase (AKS) and Methyl-parathion hydrolase (MPD) also clustered with hypothetical proteins, however, for these families of enzymes, despite also having the Metallo-hydrolase domain, it was not reported beta-lactamase activity. A hypothetical protein prospected in *E. coli* grouped with another hypothetical protein for which beta-lactamase activity has already been described in a functional characterization study, both diverge from a common origin to the beta-lactamases clade of subclass B3 [20].

As observed in the class B phylogeny, the classical class C beta-lactamase families clustered separately from the other protein families and from proteins hypothesized or annotated only as belonging to the PBP superfamily (Fig. 2C). They are part of a single clade that is divided into two groups: 1) ADC/ACC/OCH/PDC/MOX-CMY/TRU/FOX; and 2) MOR-DHA/BlaEC/AmpC/LAT-CFE-BIL-CMY/CHM/MIR-ACT-CMG-ZEG. In addition to the canonical beta-lactamases, we identified 5 other functional groups related to class C beta-lactamases: 1) AmpH (D-alanyl-D-alanine-carboxypeptidase/endopeptidase), 2) PKS (Polyketide synthases), 3) Est/PPBB, 4) EstI and 5) EstII. The hypothetical proteins, in turn, were distributed among these five main groups. The hypothetical proteins close to AmpH are divided into two groups, one closer to the sequences already characterized, which even have beta-lactamase activity, and another more distant, but with the same origin. Family VIII carboxylesterases are serine hydrolases for which beta-lactamase activity has been reported for some representatives. In our analysis, carboxylesterases formed three distinct clades, and all include representatives that exhibit promiscuous beta-lactamase activity and hypothetical proteins prospected from the genomes. Furthermore, a group of enzymes without functional classification formed a clade separate from the other three enzyme families.

### Conservation of the catalytic site between classical beta-lactamases and homologous proteins

**Fig. 3:**
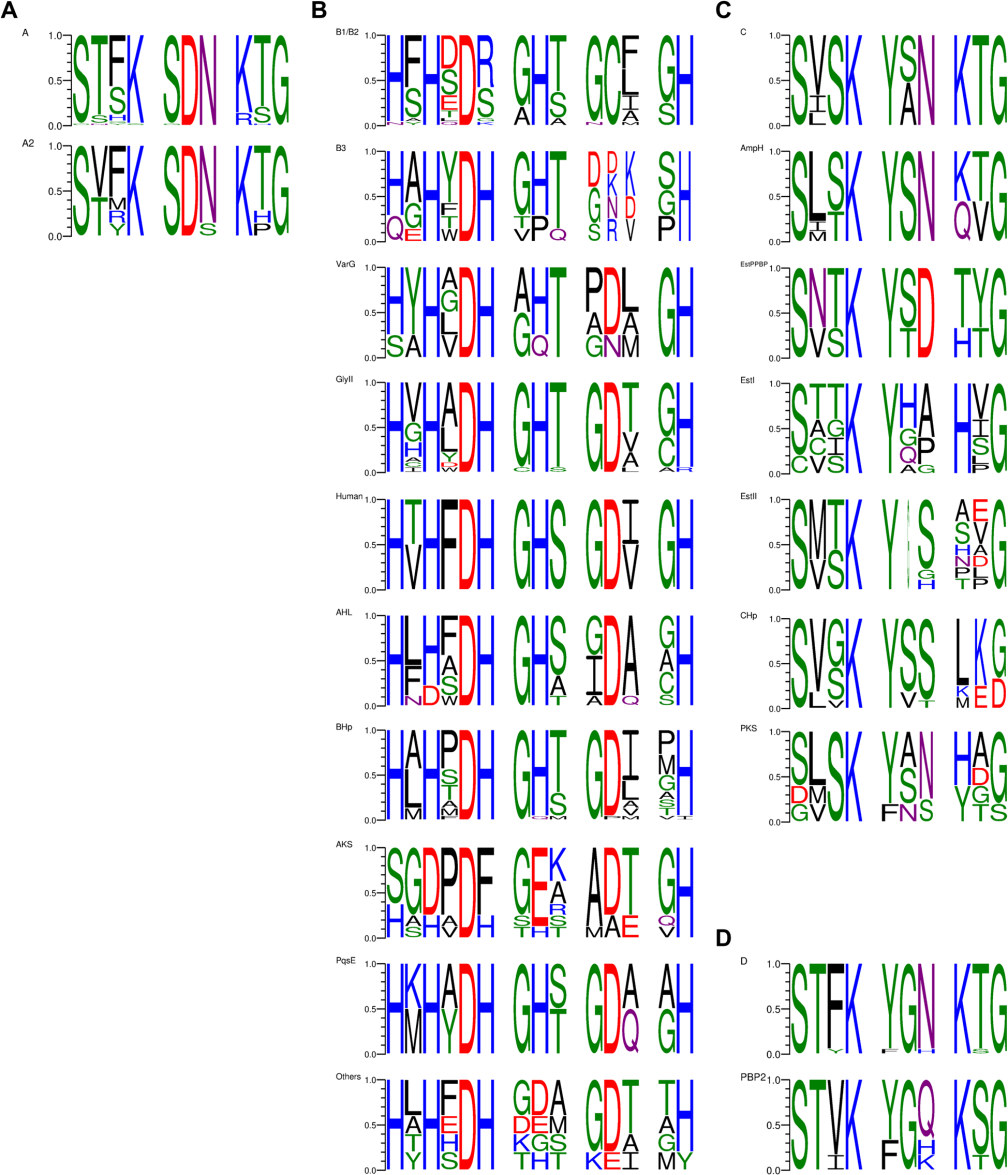
Conservation of catalytic site motifs by class and phylogenetic group of beta-lactamases. Protein catalytic site motifs were recovered and divided according to phylogenetic grouping. **A** A: class A beta-lactamases, A2: beta-lactamases and hypothetical proteins from phylogenetic group A2. **B** B1/B2: beta-lactamases of subclasses B1 and B2, B3: beta-lactamases of subclass B3, VarG: putative metallo-beta-lactamases and chlorothalonyl dehalogenase, GlyII: hypothetical proteins, ribonuclease, glyoxalases II and sulfur dioxygenase, Human: hypothetical protein and Human MBL, AHL: hypothetical proteins and N-acyl homoserine lactone hydrolases, BHp: hypothetical proteins, AKS: hypothetical proteins and alkylsulfatase, PqsE: Pseudomonas quinolone signal response proteins, Others: other functional groups. **C** C—class C beta-lactamases, AmpH: hypothetical proteins and carboxylpeptidases, Est/PPBP: carboxylesterase VIII and putative penicillin binding proteins, EstI: hypothetical proteins and carboxylesterases VIII, EstII: hypothetical proteins and carboxylesterases VIII, CHp: proteins hypothetical, PKS: hypothetical proteins and polyketide synthases. **D** D: class D beta-lactamases, PBP2: penicillin binding proteins 2

The conserved beta-lactamase-like domains identified in the prospected sequences were shown to encompass catalytic functions that go beyond beta-lactamase activity. Therefore, we evaluated the conservation of the amino acid residues that make up the catalytic site motifs for each of the four sets of proteins and their potential functional impact due to their similarity or not with classical beta-lactamases. As the distribution of proteins without functional classification was quite dispersed, especially in class B and C phylogenies, the catalytic motifs were evaluated according to the constituent proteins of the phylogenetic groups (Fig. 3).

The catalytic site of serine beta-lactamases is arranged based on three conserved or semi-conserved catalytic motifs: SxxK, [S/Y]xK and [KR]xG. In general, the prospected class A sequences presented the catalytic motifs already described as characteristic of class A beta-lactamases (SxxK SDN [KR][TS]G) [30]. The comparison of the motifs of the phylogenetic group A2 in relation to the other subfamilies did not show major differences (Fig. 3A) and the amino acid variability occurs precisely in the semi-conserved positions. The hypothetical proteins without subfamily classification, phylogenetically allocated to the A2 clade (Fig. 2A), have variations in positions considered to be conserved in the motifs. In the case of the hypothetical protein, the third motif has a histidine instead of threonine or serine (STRK SDN KHG), in the second case, the second motif has a second serine, and the third motif has a proline instead of threonine (STFK SDS KPG).

The set of class D prospected sequences includes the beta-lactamases of the OXA subfamily and PBP2. Considering the catalytic site motifs, both groups are quite similar (Fig. 3D). Differences are observed only in the proportions of certain amino acids in certain positions and, when the amino acids are different, the chemical characteristic was maintained. The most significant variation between the two types of proteins occurs in motif two, where canonical beta-lactamases have an asparagine ([Y/F]GN) while PBP2 vary between glutamine, histidine and lysine ([Y/F]G[Q/H/K]).

Like classes A and D, class C beta-lactamases are serine hydrolases whose catalytic site is arranged around three motifs (SxSK YxN KTG) [32]. Among the phylogenetic groups of the proteins analyzed, greater conservation in the composition of these motifs is observed, precisely in the group formed by the classic beta-lactamases of class C (SxSK Y[SA]N KTG) and in the proteins of the AmpH group (Sx[ST] K YSN [KQ][TV]G) (Fig. 3C). In the AmpH group the most significant difference is in the amino acid residues lysine and threonine in the third motif. In four hypothetical proteins of the AmpH subclade those residues were replaced by glutamine and valine (S[LI]SK YSN QVG). Proteins functionally classified as esterases, showed greater variation in the 3 motifs, maintaining the conservation of 4 residues (SxxK Yxx xxG). The proteins from the CHp and PKS groups presented fewer conserved residues in relation to the canonical motifs (Fig. 3C).

Class B metallo beta-lactamases were those with the highest diversity of homologous proteins. This feature is also reflected in the variety of motifs within the catalytic site, of concerning the semi conserved residues. The catalytic site of MBLs is mainly composed of histidines that act as ligands for zinc ions, since the proteins of this superfamily are metallo hydrolases. The residues already well characterized as essential in subclasses B1/B2 ([HN]xHxDx xHx xCx xH) differ little from those in subclass B3 ([HQ]xHxDxH xHx xxx xH) [31], and corroborate what we found in the proteins of those subclasses prospected from the genomes (Fig. 3B). Other points worth mentioning: the first motif of the catalytic site of class B1/B2 beta-lactamases has two conserved histidines, while B3 and all other homologous proteins have three highly conserved histidine residues; in all groups of homologous proteins there is an apparent conservation of an aspartate residue in the third motif, a position that in the B1/B2 subclasses is occupied by a cysteine residue (Fig. 3B

### Association between beta-lactamases homologous proteins and the minimum inhibitory concentration for beta-lactam antibiotics

The bacterial strains whose genomes were used in this study have phenotypic information determined through laboratory testing for resistance to beta-lactam antibiotics, represented by the MIC value for the antibiotic. Using this information, we investigated the association between proteins homologous to beta-lactamases and resistance phenotype groups. We selected the antibiotics imipenem (Ip), meropenem (Mp), ceftazidime (CefT), cefazolin (CefZ), ampicillin (Amp), and aztreonam (Az), covering the four classes of beta-lactams. For each antibiotic, we identified genomes with available MIC values. Due to the rare occurrence of some MIC values (Supplementary Fig. 1A), we categorized these values into three resistance phenotype groups: S) susceptible; R) resistant (MIC greater than or equal to 8 and less than 32 mg/L); and R2) resistant (MIC greater than or equal to 32 mg/L) (Supplementary Fig. 1B).

The Fisher exact test revealed association between the presence of classical and homologous proteins and the resistance phenotype categories for most of the phylogenetic groups (Supplementary Table S4). The homologous protein groups AHp, GlyII, PBP2, and the B3 beta-lactamase group were the only ones that did not show an association with any antibiotic. Despite being a classical beta-lactamase, the lack of association of the B3 group with antibiotics is likely due to the low frequency of these proteins in genomes, as well as the hypothetical proteins in the group. Despite their close phylogenetic proximity to the classical enzymes (Fig. 2B), they are unlikely to be functional.

**Fig. 4:**
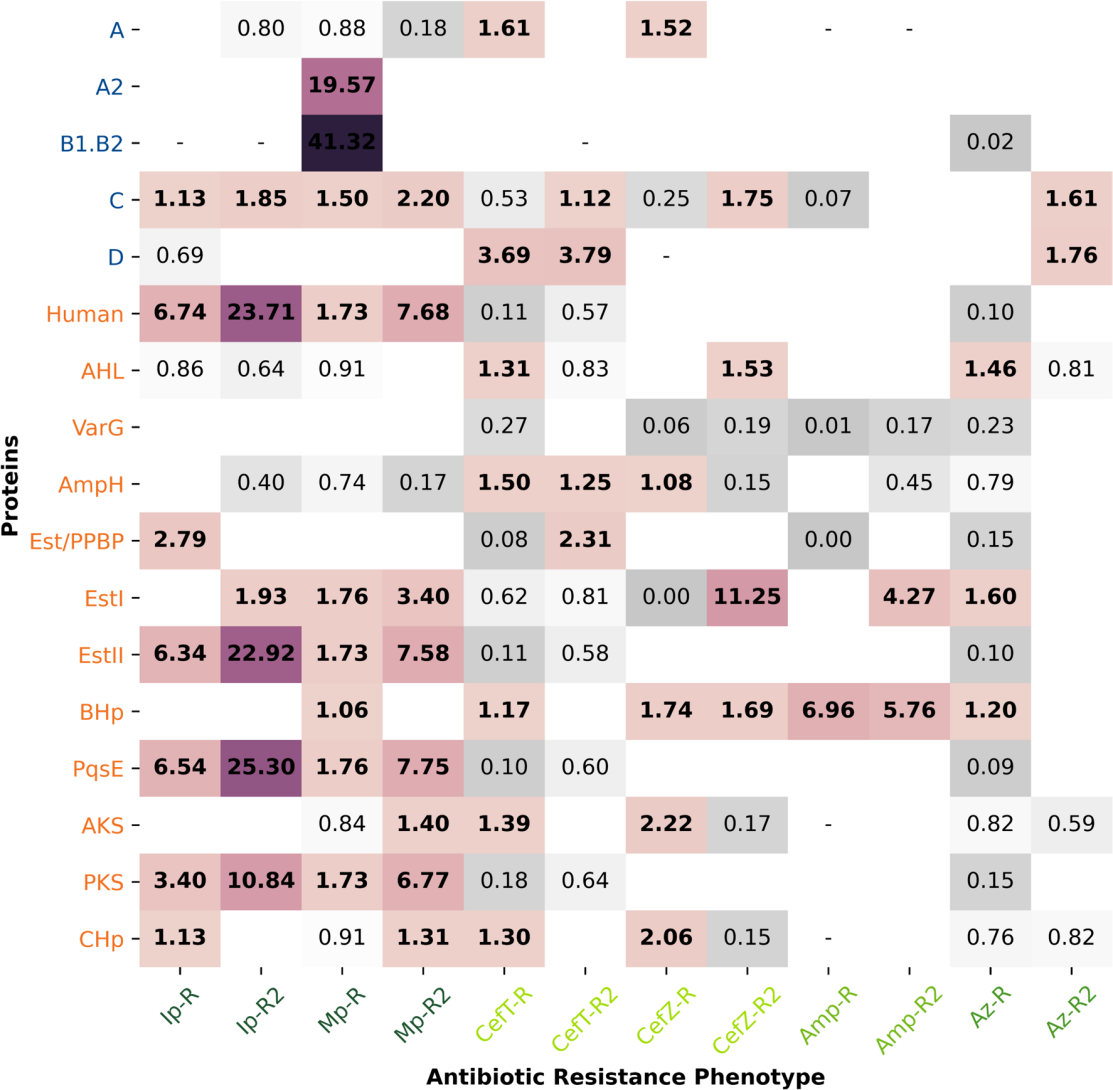
Relative risk for the resistant R and R2 categories in relation to the sensitive category. Ip—imipenem, Mp—meropenem, CefT—ceftazidime, CefZ—cefazolin, Amp—ampicillin, Az—aztreonam. Comparisons that did not show statistical differences in the paired Fischer test were omitted. Positions where relative risk calculation was not possible due to frequency 0 in the sensitive category are indicated by a dash (-). beta-lactamases are marked in blue and homologous proteins are marked in orange. We highlight in bold comparisons with RR greater than 1

For those protein groups showing statistical significance, we compared the differences between the resistant phenotypes R and R2 with the susceptible phenotype (Supplementary Table S5) in order to identify whether protein groups are more prevalent in antibiotic-resistant organisms. In addition to classical beta-lactamases, some groups of homologous proteins also showed an increased frequency in the resistance categories compared to the susceptible one (Fig. 4). The carbapenems Ip and Mp exhibited the highest frequency of increased protein cases in R and R2. In addition to classical beta-lactamases from classes A2 and B1/B2 in Mp-R and class C in Ip-R, Ip-R2, Mp-R, and Mp-R2, the Human, EstII, PqsE, and PKS groups also showed a higher frequency in both resistance categories for the two carbapenems. Furthermore, a higher frequency was also observed in Ip-R for Est/PPBP and CHp, in Ip-R2 for EstI, in Mp-R for EstI and BHp, and in Mp-R2 for EstI, AKS, and CHp. The A, AHL, and AmpH groups are more frequent in genomes sensitive to carbapenems than in resistant ones. The classical beta-lactamases A, C, and D are more frequent in genomes with CefT-R and CefZ-R phenotypes, CefT-R2 and CefZ-R2, and CefT-R and CefT-R2, respectively. Unlike carbapenems, the Human, EstI, EstII, PqsE, and PKS groups are less frequent in terms of resistance to CefT. The AHL, AmpH, BHp, AKS, and CHp groups have increased frequency in CefT-R, Est/PPBP in CefT-R2, CHp, AKS, and BHp in CefZ-R, AmpH in CefT-R2 and CefZ-R, and EstI in CefZ-R2.

In general, the resistant groups for Amp and Az do not present higher frequencies for the presence of protein groups when compared to the susceptible one (RR greater than 1). Nonetheless, the BHp group is more frequent in Amp-R, Amp-R2 and Az-R, the EstI group is more frequent in Amp-R2 and Az-R and the AHL group in Az-R. Class C and D beta-lactamases are more frequent in Az-R2. All groups of homologous proteins, except EstI and BHp, and beta-lactamases B1/B2 are less frequent in Az-R. Except for carbapenems, CefT-R2 and Az-R2, for which there was no statistical significance, the VarG group is less frequent in all other resistance groups.

## Discussion

Beta-lactamases are a large group of enzymes and one of the main factors contributing to resistance to beta-lactam antibiotics. These enzymes are of particular concern in gram-negative bacteria and have contributed to a critical scenario of infections caused by multidrug-resistant organisms that are difficult to treat [5, 6, 10]. In this study, we aimed to identify beta-lactamases homologous proteins that may be contributing to resistance to beta-lactam antibiotics in clinically relevant gram-negative bacteria: *K. pneumoniae*, *A. baumannii*, *P. aeruginosa*, *Enterobacter* spp., and *E. coli*. The prospecting method retrieved a considerable number of beta-lactamases and homologous proteins. The average number of proteins identified per genome, related solely to resistance to beta-lactam antibiotics, was equal to or greater than seven for all species. This significant accumulation of resistance genes to only one class of antibiotics is concerning and has become increasingly common, especially in clinical isolates [6, 35].

In both the construction of profiles and the filtering of prospect proteins, the presence of protein domains was assessed using Pfam identifiers. Except of class A, the domains found as characteristic of each of the four sequence groups were found to encompass not only beta-lactamase subfamilies but also other functional groups. This is related to the fact that serine and metallo-beta-lactamases belong to the Penicillin-binding-protein-like (PBP-like) and Metallo-beta-lactamase (MBL) superfamilies, which encompass different functional groups that share the same remote phylogenetic origins and a similar domain arrangement [18]. The majority of subfamilies present in the genomes were identified in more than one species (Fig. 1), which is quite common for resistance genes due to their ability to undergo horizontal transfer between species [36].

Apparently, the domain of class A beta-lactamases is highly conserved among themselves and divergent from other protein domains, as only one hypothetical protein was prospected along with representatives of the canonical subfamilies of class A. Phylogenetically, class A proteins are divided into groups A1 (subgroups A1a, A1b, and A1c) and A2 [30]. The analyzed genomes have representatives in all phylogenetic subgroups (Fig. 2A). It has been reported that the A2 group encompasses a set of potential subfamilies due to the grouping of many uncharacterized proteins in this clade [18, 33], as also observed in our analysis. The already known subfamilies of this group (PER/VEB/TLA/CME) are extended-spectrum cephalosporinases [13]. Despite its close phylogenetic relationship with the cephalosporinases in the A2 group, the hypothetical protein differs from them in the final catalytic motif and did not show an association with any beta-lactam class.

PBP2, which shares the transpeptidase domain (pfam00905) with canonical class D beta-lactamases, are high molecular weight (HMW) PBPs. PBPs are transpeptidases or carboxypeptidases involved in the synthesis of bacterial cell wall peptidoglycan. These enzymes have motifs and a catalytic site structure similar to serine beta-lactamases and are the target site for beta-lactam antibiotics. They operate with the same acylation mechanism as canonical serine beta-lactamases, where the nucleophilic serine attacks the carbonyl of the beta-lactam ring, forming the acyl-enzyme complex. However, unlike beta-lactamases, which have acquired the ability to rapidly hydrolyze this intermediate, in PBPs, this complex is covalent and hydrolyzes very slowly, preventing further reactions [18, 37]. PBP2s have very similar catalytic motifs (Fig. 3D) but are phylogenetically distant from class D beta-lactamases (Fig. 2D). Although they did not show an association with the evaluated antibiotics, it has been previously reported that mutations in the *mrdA* gene, which encodes PBP2, led to an increase in the MIC for imipenem and carbapenem in *E. coli* [38, 39].

The significant number of proteins that were prospectively identified and are functionally undescribed ('hypothetical' or 'putative') reflects an ongoing and recurrent issue in protein identification [14, 40]. When evaluating these sequences in the phylogenetic trees of classes B and C, we were able to identify that, despite the distance to the classical beta-lactamases, many of them are close to other groups of the MBL and PBP-like families (Fig. 2B and C). Furthermore, studies have reported that enzymes from the metallohydrolase and serine hydrolase superfamilies, but functionally distinct from classical beta-lactamases, can be bifunctional, exhibiting a certain degree of beta-lactamase activity, such as carboxylesterases VIII [41, 42] e glyoxalases II [20, 43].

The MBL superfamily encompasses a large number of metalloenzymes distributed across the three domains of life, sharing structural homology and conserved histidine motifs responsible for metal ion binding. These enzymes perform various functions such as hydrolases, lactonases, and RNases. Canonical class B beta-lactamases constitute only 1.5% of the proteins in this superfamily and have two distinct phylogenetic origins [44], this indicates that zinc-dependent beta-lactam hydrolysis evolved independently in the B1/B2 and B3 groups [45]. Moreover, enzymatic promiscuity for beta-lactamase activity spans nearly all branches of the phylogenetic tree of class B beta-lactamases. This promiscuous activity has been identified and experimentally determined in the GlyII, Human MBL, AHL, and VarG subfamilies, which are groups where hypothetical proteins have clustered (Fig. 2B). The diversity of homologous proteins found reflects the diversity of the proteins in the metallo-beta-lactamase superfamily, whose representatives have varied functions, but present sequence homology and similar catalytic domain topology. In terms of function, canonical class B beta-lactamases are highly specialized and efficient in the degradation of beta-lactam antibiotics, especially carbapenems. For the homologous proteins identified that present promiscuous beta-lactamase activity in addition to their primary functions, the primary activity tends to be highly efficient, whereas the promiscuous activity is less efficient when compared to that observed in specialized beta-lactamases from class B.

The MBLAC2 protein belongs to the group of 18 MBLs already identified in humans. Some of these, including MBLAC2, exhibit beta-lactamase activity, but the majority are still poorly characterized regarding their canonical function [46, 47]. The group phylogenetically close to MBLAC2 is found only in *P. aeruginosa* (Fig. 2A) and has been shown to be more prevalent in genomes resistant to carbapenems, especially in Ip-R2, where it is 23.71 times more frequent when compared to sensitive cases.

The AHL group proteins include hypothetical proteins and N-acyl-homoserine lactone hydrolases, which are quorum-quenching proteins [48]. They are closely related to 4-pyridoxolactonase, a protein from *Mesorhizobium loti* that exhibits promiscuous beta-lactamase activity [20, 49]. Despite this, the frequency of the AHL group in resistant genomes is not consistently high, as in some cases, such as Ip, Mp, CefT-R2, and Az-R2, the presence rates are lower than in the sensitive group.

The VarG protein was identified in *Vibrio cholerae* and is highly specific for the hydrolysis of carbapenems. Based on this, it has been proposed that this protein could indicate the existence of a new subclass of MBL since it is phylogenetically close to subclasses B1/B2 but belongs to another clade [18, 44, 50]. Our results indicate that VarG may not be restricted to *V. cholerae* but is dispersed in other bacterial species of clinical interest. Despite the phylogenetic proximity, the conservation of the catalytic motif (Fig. 3B), and the association with genomes resistant to carbapenems were not significant. Additionally, it is more prevalent in genomes sensitive to cephalosporins, ampicillin, and aztreonam (Fig. 4).

The other homologous class B groups, annotated as BHp, PqsE and AKS, do not have previously reported beta-lactamase activity. For the AKS group, which includes hypothetical proteins and putative akyl/aryl sulfatases (Fig. 3B), we did not identify a consistent higher frequency of presence in resistant genomes (Fig. 4 and Supplementary Table S5). Pseudomonas quinolone signal response proteins (PqsE) are quorum-sensing proteins, which can also act to mediate iron acquisition, cytotoxicity, vesicle biogenesis and immunomodulation [51], which have been shown to be more frequent in carbapenem-resistant genomes (Fig. 4). The BHp group, which includes only hypothetical proteins distributed among all species, has a higher frequency of presence in resistant genomes for all antibiotics in at least one of the two resistance categories (Fig. 4), especially in ampicillin, with RR equal to 6.96 and 5.76 for the Amp-R and Amp-R2 groups, respectively. Results like these demonstrate the existence of an exploitable potential regarding the dispersion and diversity of this group of enzymes, which have not yet been completely elucidated.

Similar situation is observed with class C enzymes, where canonical subfamilies are phylogenetically distant from others; however, enzymatic promiscuity is observed in groups with carboxylesterases VIII and AmpH (Fig. 2C). AmpH are low molecular weight (LMW) PBPs very close phylogenetically and in sequence identity (~ 30%) to the chromosomal beta-lactamase AmpC. Therefore, it is proposed that class C beta-lactamases originated from a structural rearrangement of LMW-PBPs of the AmpH type [18]. Not all enzymes in this group exhibit beta-lactamase activity, but weak activity against the nitrocefin chromogenic cephalosporin substrate has been observed [52]. The relative risk analysis corroborates with these previous findings, where for CefT, we observed an increased frequency in the resistant groups, which did not extend to CefZ (RR = 0.15, indicating higher frequency of the presence of the protein group in the susceptible category) (Fig. 4).

Unlike other carboxylesterases that have the catalytic serine in the GxSxG motif, carboxylesterase group VIII has the same SxxK motif as beta-lactamases. Many studies have evaluated beta-lactams as substrates for carboxylesterases VIII, and most of them show activity against nitrocefin and cephalosporins of varying spectrum [53–55]. However, it is still not completely elucidated in which region of the molecule these enzymes act. In some cases, they cleave the beta-lactam ring in the same way as beta-lactamases [56], but in others, cleavage occurs in ester bonds, keeping the beta-lactam ring intact [54, 57]. However, the proteins identified in genomes that are close to group VIII carboxylesterases (Est/PPBP, EstI, and EstII) did not show a clear increased frequency in genomes resistant to cephalosporins. However, all of them are more frequent for carbapenems, and EstI is more frequent in Amp-R2 and Az-R. The PKS and CHp groups did not cluster with representatives that had promiscuous beta-lactamase activity. For the CHp group, no consistently increased frequency was observed in the two resistance groups for the antibiotics we analyzed. The PKS group, like others frequent in *P. aeruginosa* (Human and PqsE), was frequent in genomes resistant to carbapenems.

### Final considerations

In this study, we have demonstrated the presence of the beta-lactamase-like domain in various proteins belonging to the PBP-like and MBL superfamilies. A significant number of the identified proteins can be associated with one of the four canonical molecular classes of beta-lactamases, emphasizing the relevance of bacteria carrying them in the antimicrobial resistance scenario. Despite hypothetical proteins not displaying characteristics of beta-lactamases, they are closely related to homologous families that may exhibit some degree of activity against beta-lactam antibiotics beyond their known functions. Enzymatic promiscuity is a way to discover evolutionary relationships among enzymes, as the emergence of new functions is more likely through the optimization of promiscuous activity [18]. These homologous enzymes may not be explicitly determinants of the resistance phenotype, but their association with canonical beta-lactamases and the selective pressure from the widespread use of beta-lactam antibiotics may favor the eventual optimization of these functions.

### Supplementary Information

Supplementary Material 1.

Supplementary Material 2.

Supplementary Material 3.  

Supplementary Material 4. 

## Data Availability

The datasets generated during and/or analysed during the current study are available in the Github repository, https://github.com/joycedesouza/BL_Homologous.

## Abbreviations

AMR: Antimicrobial resistance
HMM: Hidden Markov Model
BV-BRC: Bacterial and Viral Bioinformatics Resource Center
MIC: Minimum inhibitory concentration
MBL: Metallo-beta-lactamase
AHL: N-acyl homoserine lactone hydrolases
PqsE: Pseudomonas quinolone signal response proteins
AKS: Alkyl/aryl-sulfatase
PKS: Polyketide synthases
PBP: Penicillin-biding proteins
PBP2: Penicillin-biding protein 2
RNase: Ribonucleases
GlyII: Glyoxalase II
SDO: Sulfur dioxygenase
MBLAC2: Human metallo-beta-lactamase
MPD: Methyl-parathion hydrolase
Est: Esterase
Mp: Meropenem
Ip: Imipenem
CefT: Ceftazidime
CefZ: Cefazolin
Amp: Ampicillin
Az: Aztreonam
