# Supplementary material for: Potential involvement of beta-lactamase homologous proteins in resistance to beta-lactam antibiotics in gram-negative bacteria of the ESKAPEE group
Source: BMC Genomics. 2024 May 22;25:508. doi: 10.1186/s12864-024-10410-2 (PMC11112869; doi:10.1186/s12864-024-10410-2)
Supplement: Supplementary file 1 — Supplementary Material 1. [file 12864_2024_10410_MOESM1_ESM.pdf]

Supplementary material

Potential Involvement of b-lactamase homologous proteins in resistance to b-lactam antibiotics in gram-negative bacteria of the ESKAPEE group

Joyce de Souza[1], Alexandre Zanatta Vieira[1], Hellen Geremias dos Santos[2], Helisson Faoro[1,3,*]

[1]Laboratory for Applied Science and Technology in Health, Carlos Chagas Institute, FIOCRUZ, Paraná, 81350-010, Brazil

[2]Carlos Chagas Institute, FIOCRUZ, Paraná, 81350-010, Brazil

[3]CHU de Quebec Research Center, Department of Microbiology, Infectious Disease and Immunology, University Laval, Quebec, QC G1V 0A6, Canada.

*Correspondence: helisson.faoro@fiocruz.br, helisson.faoro@crchuldequebec.ulaval.ca; Tel.: +1 (418) 525-4444

Figure S1. Distribution of minimum inhibitory concentration (MIC) for antibiotics. Ampicillin (Amp), Aztreonam (Az), Ceftazidime (CefT), Cefazolin (CefZ), Imipenem (Ip), Meropenem (Mp). (A) Distribution of MIC values between antibiotics. (B) Distribution of sensitive (1), resistant (2) and resistant R2 (3) MIC categories.

A

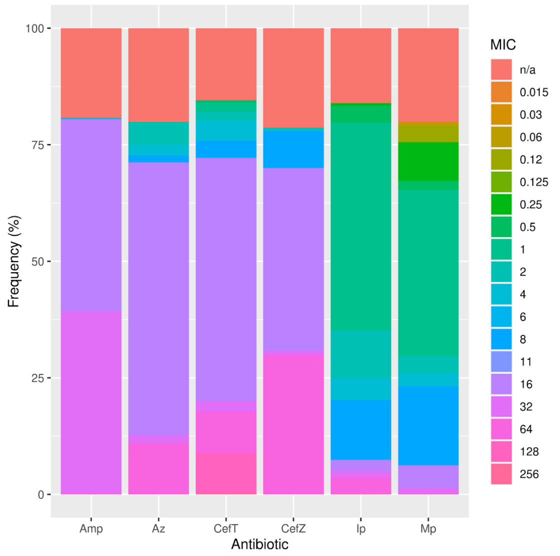

B

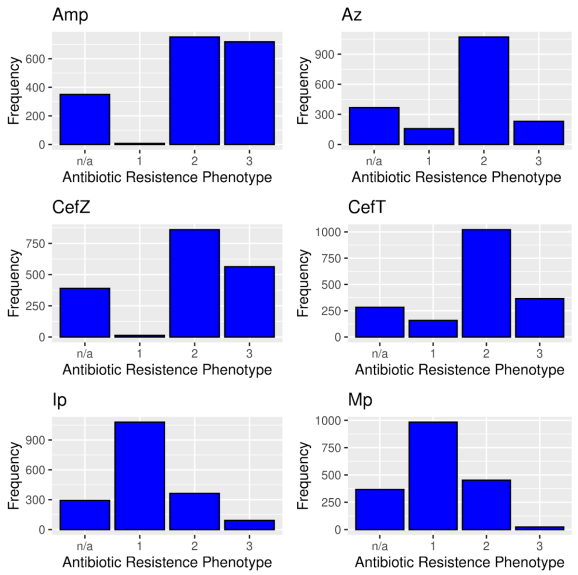

Amp (1476)
1 - [2, 4, 8] mg/L
2 - [16] mg/L
3 - [32, 64] mg/L

CefZ (1436)
1 - [1, 2, 4] mg/L
2 - [8, 16] mg/L
3 - [32, 64] mg/L

Ip (1533)
1 - [0.25, 0.5, 1, 2] mg/L
2 - [4, 8, 16] mg/L
3 - [32, 64, 256] mg/L

Az (1459)
1- [1, 2, 4, 8] mg/L
2 - [11, 16] mg/L
3 - [32, 64] mg/L

CefT (1544)
1 - [0.5, 1, 2, 4] mg/L
2 - [6, 8, 16] mg/L
3 - [32, 64, 128, 256] mg/L

Mp (1459)
1 - [0.015, 0.03, 0.06, 0.12, 0.125, 0.25, 0.5, 1,2] mg/L
2 - [4, 8, 11, 16] mg/L
3 - [32, 64, 128] mg/L

Table S2. Proteins from other functional groups of the MBL superfamily included in the class B phylogeny

ID		Activity	Reference	BL act
PTS	A0A411PXC0	Phytase	https://doi.org/10.1128/msphere.00167-19	Yes
PRNase	A0A6L8PRK4, Q81U06	Putative ribonuclease	http://dx.doi.org/10.1016/j.jmb.2014.04.013	No
Igni18	A8ABX8	Promiscuous ancestral enzyme	https://doi.org/10.1038/s42003-021-01671-8	Yes
Chd	C9EBR5	Chlorothalonil dehalogenase	http://dx.doi.org/10.1016/j.jmb.2014.04.013	Yes
GlyII	O24495	Glyoxalase II	https://doi.org/10.1021/bi9010539	Yes
AHL	P0CJ63	AHL-lactonase	http://dx.doi.org/10.1016/j.jmb.2014.04.013	No
ARS	P28607	Arylsulfatase	http://dx.doi.org/10.1016/j.jmb.2014.04.013	No
GlyII	Q16775	Glyoxalase II	http://dx.doi.org/10.1016/j.jmb.2014.04.013	No
MBLAC2	Q68D91	Human MBL	https://doi.org/10.1038/s41598-019-48723-y	Yes
MPD	Q841S6	Methyl-parathion hydrolase	http://dx.doi.org/10.1016/j.jmb.2014.04.013	No
PCE	Q8DQ62	Phosphorylcholine esterase	http://dx.doi.org/10.1016/j.jmb.2014.04.013	No
AHL	Q988B9	4-Pyridoxolactonase	http://dx.doi.org/10.1016/j.jmb.2014.04.013	Yes
SDO	Q9C8L4	Sulfur dioxygenase	http://dx.doi.org/10.1016/j.jmb.2014.04.013	Yes
AKS	Q9I5I9	Alkylsulfatase	http://dx.doi.org/10.1016/j.jmb.2014.04.013	No
GlyII	Q9SID3	Glyoxalase II	http://dx.doi.org/10.1016/j.jmb.2014.04.013	Yes
Hp	Q9X207	Hypothetical	http://dx.doi.org/10.1016/j.jmb.2014.04.013	Yes
VarG	AAF94716.1	Putative MBL	https://doi.org/10.1371/journal.pone.0184255	Yes
RNase	WP_048116343.1	Ribonuclease	https://doi.org/10.3390/life10110280	Yes

Table S3. Proteins from other functional groups of the SBL superfamily included in the class C phylogeny

	ID	Activity	Reference	BL act
EstB	AAF59826.1	Carboxylesterase	https://doi.org/10.1016/S0168-1656(01)00284-X	No
AmpH	NP_414910.1	Endopeptidase/ Carboxypeptidase	https://doi.org/10.1128/jb.05764-11	Yes
EstC	ACH88047.1	Carboxylesterase	https://doi.org/10.1007/s00253-009-1895-x	Yes
CcEstA	YP_002515630.1	Carboxylesterase	https://doi.org/10.1038/srep37978	Yes
EstM-N1	AEA07653.1	Carboxylesterase	https://doi.org/10.1007/s00253-011-3132-7	Yes
EstU1	AFU54388.1	carboxylesterase	https://doi.org/10.1002/prot.24334	Yes
Est22	AGT17593.1	Carboxylesterase	https://doi.org/10.1016/j.bbrc.2013.06.076	Yes
*Ps*EstA	AHL66978.1	Carboxylesterase	https://doi.org/10.3390/biom9120786	Yes
EstSTR1	AJE68931.1	Carboxylesterase	https://doi.org/10.1186/s40064-016-2172-y	Yes
EstM2	AJG42113.1	Carboxylesterase	https://doi.org/10.1186/s12934-020-01336-x	No
*Lg*LacI	WP_042218452.1	Carboxylesterase	https://doi.org/10.1016/j.ijbiomac.2022.02.081	Yes
EstCS3	ARE60547.1	Carboxylesterase	https://doi.org/10.1016/j.ijbiomac.2020.09.070	Yes
